# Primary Stability of Self-Drilling and Self-Tapping Mini-Implant in Tibia of Diabetes-Induced Rabbits

**DOI:** 10.1155/2014/429359

**Published:** 2014-05-11

**Authors:** Jea-Beom Park, Eun-Young Kim, Janghyun Paek, Yoon-Ah Kook, Do-Min Jeong, Il-Sik Cho, Gerald Nelson

**Affiliations:** ^1^Department of Orthodontics, The Catholic University of Korea, Seoul, Republic of Korea; ^2^Department of Dentistry, National Medical Center of Korea, 245 Euljiro, Junggu, Seoul 100-799, Republic of Korea; ^3^Department of Pediatrics, School of Medicine, Chosun University, 30-1 Hakdong, Donggu, Gwangju 501-717, Republic of Korea; ^4^Department of Prosthodontics, School of Dentistry, Kyung Hee University, Hoegidong, Dongdaemungu, Seoul 130-701, Republic of Korea; ^5^Department of Dentistry, Korea University, 80 Gurodong, Gurogu, Seoul 152-703, Republic of Korea; ^6^Division of Orthodontics, School of Dentistry, University of California San Francisco, 707 Parnasus Avenue, San Francisco, CA 94143, USA

## Abstract

*Objective*. This study aimed to evaluate effects of type 1 diabetes mellitus and mini-implant placement method on the primary stability of mini-implants by comparing mechanical stability and microstructural/histological differences. *Methods*. After 4 weeks of diabetic induction, 48 mini-implants (24 self-tapping and 24 self-drilling implants) were placed on the tibia of 6 diabetic and 6 normal rabbits. After 4 weeks, the rabbits were sacrificed. Insertion torque, removal torque, insertion energy, and removal energy were measured with a surgical engine on 8 rabbits. Remaining 4 rabbits were analyzed by microcomputed tomography (micro-CT) and bone histomorphometry. *Results*. Total insertion energy was higher in self-drilling groups than self-tapping groups in both control and diabetic groups. Diabetic groups had more trabecular separation in bone marrow than the control groups in both SD and ST groups. Micro-CT analysis showed deterioration of bone quality in tibia especially in bone marrow of diabetic rabbits. However, there was no statistically significant correlation between self-drilling and self-tapping group for the remaining measurements in both control and diabetic groups. *Conclusions*. Type 1 diabetes mellitus and placement method of mini-implant did not affect primary stability of mini-implants.

## 1. Introduction

Primary stability is the most important factor for the survival of mini-implants and is obtained by mechanical interlock between bone and mini-implant [1–3]. Most of orthodontic mini-implants are either self-tapping (ST) or self-drilling (SD) type. A ST mini-implant requires predrilling and a SD mini-implant is placed without predrilling [4–7]. The ST system has been used for a long time but complications can occur during predrilling such as thermal damage, root damage, and a drill fracture.

Placement of the SD mini-implant is simple and takes less time and thermal damage can be avoided. Moreover, there is no risk of the drill fracture. This system also enhances primary stability by compressing bones during implantation and contact surface of bone to implant is wider [4]. Therefore, primary stability of SD mini-implants is affected by intimate bony contact during placement. Micromobility of a mini-implant is minimized proper stability, which enhances new bone formation [8]. The SD system is advantageous with better stability, especially in bone with low density such as maxilla and adolescent patients [6, 7, 9, 10]. In high density bone or thick cortical bone, however, the SD system is disadvantageous in obtaining good primary stability by inducing excessive pressure that can cause microfracture, adjacent cell damage, and other complications [5].

Majority of orthodontic patients are healthy young individuals. But there has been dramatic increase in the number of adults seeking orthodontic treatment in the last 20 years. And more diabetic patients are getting orthodontic treatment. According to the U.S. Department of Health and Human Services, 15,600 young people (younger than 20 years of age) were newly diagnosed with type 1 diabetes annually in the United States during 2002–2005. Therefore, clinicians are required to understand effects of diabetes in orthodontic treatment, especially when placing mini-implants.

Although it is still controversial, diabetes is known to disrupt blood supply by inducing microvascular complications and delay wound healing and increase susceptibility to infection and periodontal disease [11, 12]. It was reported that the stability of mini-implant was not obtained in diabetic patients because of poor bone quality and chronic hyperglycemia, which suppressed osteoblast differentiation [13]. Many previous literatures reported that mineral metabolism and formation of osteoid and bone were reduced in diabetic hyperglycemic state [14].

The SD system, which can enhance bone-implant contact by compressing bone, is advantageous to obtain good primary stability. However, it has not been studied how the SD mini-implant affects stability in diabetic patients. The purpose of this study was to evaluate effects of type 1 diabetes mellitus (DM) and mini-implant type (self-drilling and self-tapping) on primary stability of mini-implants. Comparative analysis between intentionally induced diabetic rabbits and control group was performed 4 weeks after placement in mechanical stability and microstructural/histological differences.

## 2. Materials and Methods

### 2.1. Animals

Six healthy controls and six diabetic New Zealand white rabbits were used in this study. Mini-implants were placed 4 weeks after diabetic induction. Those mini-implants were removed in 4 control rabbits and 4 diabetic rabbit following 4 weeks of healing. Micro-CT and histological analysis were performed in four rabbits. The experiment protocol was approved by the Institutional Animal Care and Use Committee. (CUMC-2010-0093-01).

For the alloxan injection, rabbits were lightly anesthetized with ketamine hydrochloride 30 mg/kg and xylazine 3 mg/kg (IM). Alloxan monohydrate (Sigma Aldrich Chemical, Saint Louis, MO, USA) was dissolved in sterile normal saline to achieve concentration of 5% (W/V), and 100 mg/kg was immediately administered intravenously via marginal ear vein for 2 minutes. 4, 8, and 12 hours after alloxan injection, 10 mL of glucose (5% W/V) was administered subcutaneously [15]. Four days after the injection, blood samples were collected from aural vein and blood glucose level was monitored using ACCU-CHECK Performa (Roche Diagnostics, Mannheim, Germany). If glucose level was over 200 mg/dL, diabetes was diagnosed [16]. Blood glucose level and weight of rabbits were also monitored weekly to check the diabetic state.

### 2.2. Surgical Procedures for the Implantation of Orthodontic Mini-Implant

**Figure 1:**
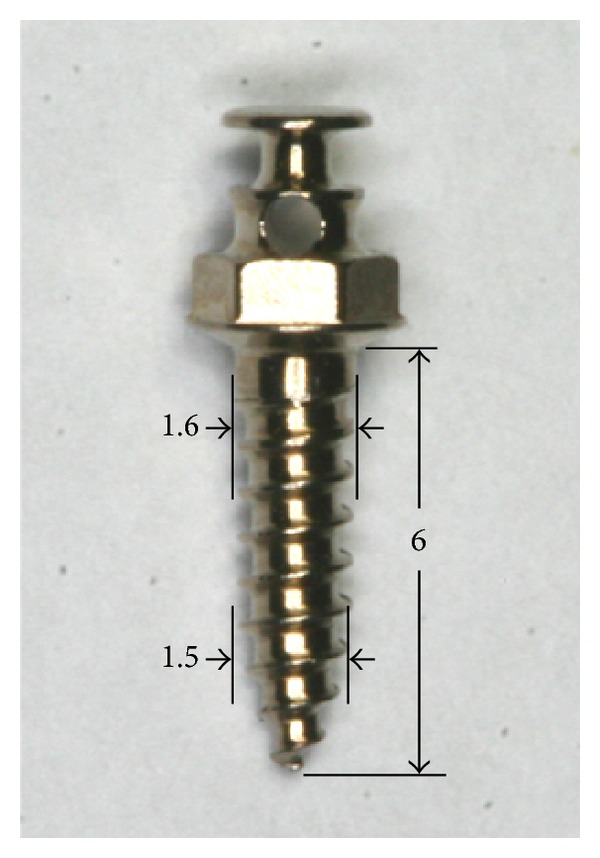
Orthodontic mini-implant used for this study. The size is 1.6 mm at the neck and 1.5 mm at the apex in external diameter and 6 mm in length (Jin-E Screw, Jin Biomed Co., Bucheon, Korea).

**Figure 2:**
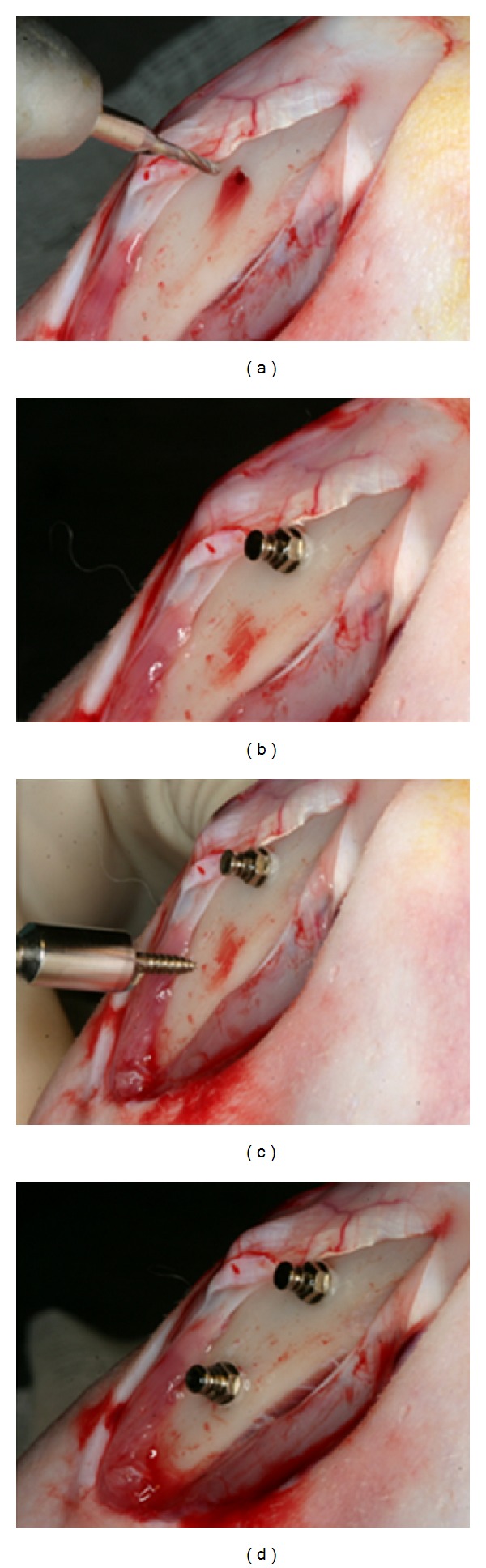
Orthodontic mini-implants implanted in rabbit tibia. (a) Predrilling for the self-tapping mini-implant was perfomed. (b) Self-tapping mini-implant was placed into the hole. (c), (d) Self-drilling mini-implant was placed into the bone without predrilling.

Orthodontic mini-implants (Ti-grade-V-alloy, Jinbiomed, Bucheon, Korea) used in this study were modified cylindrical type (external diameter: 1.6 mm at neck, 1.5 mm at apex, length: 6 mm) with cutting flutes at the apex (Figure 1). Two implants in one tibia were placed and a total of 4 implants were placed on each rabbit. Forty-eight implants (24 for diabetic group and 24 for control group) were placed. The self-tapping (ST) mini-implants were placed after predrilling with 1.0 mm diameter pilot drill (Figure 2). Implants position was assigned by complete random block design. Depth of placement was up to smooth surface below the threaded end of mini-implant head. A surgical engine (Elcomed SA-200C, W&H, Burmoos, Austria) was used to record torque value in every 0.125 second during insertion and removal of mini-implants. After the surgery, flap was closed. Analgesics (Ketoprophen 1 mg/kg, q.d.) and antibiotics (Gentamicin—4 mg/kg, q.d.) were subcutaneously administered for 3 days. The rabbits were sacrificed following four weeks of healing using an overdose of anesthetics to induce heart failure.

### 2.3. Micro-CT and Histomorphometrical Analysis

**Figure 3:**
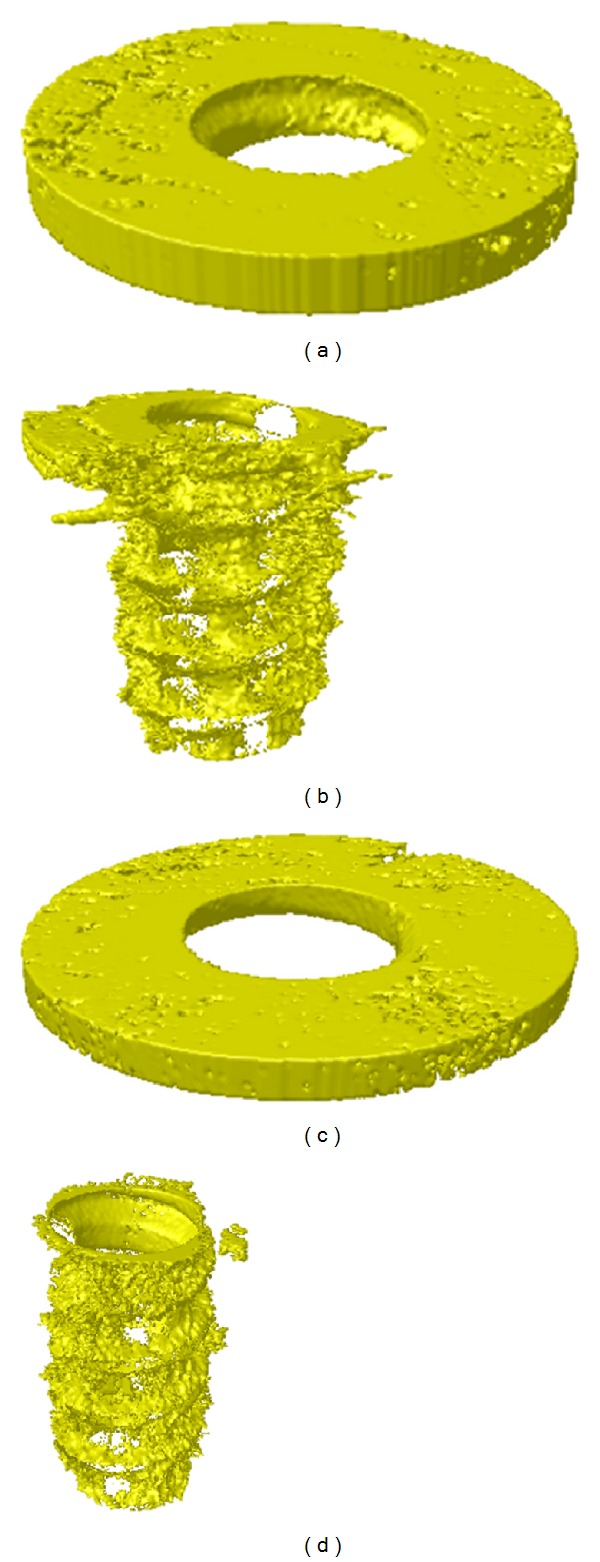
3D micro-CT images of bone microarchitecture determined by the volume of interest (VOI). (a) Cortical bone area in control group. (b) Bone marrow area in control group. (c) Cortical bone area in diabetic group. (d) Bone marrow area in diabetic group.

**Figure 4:**
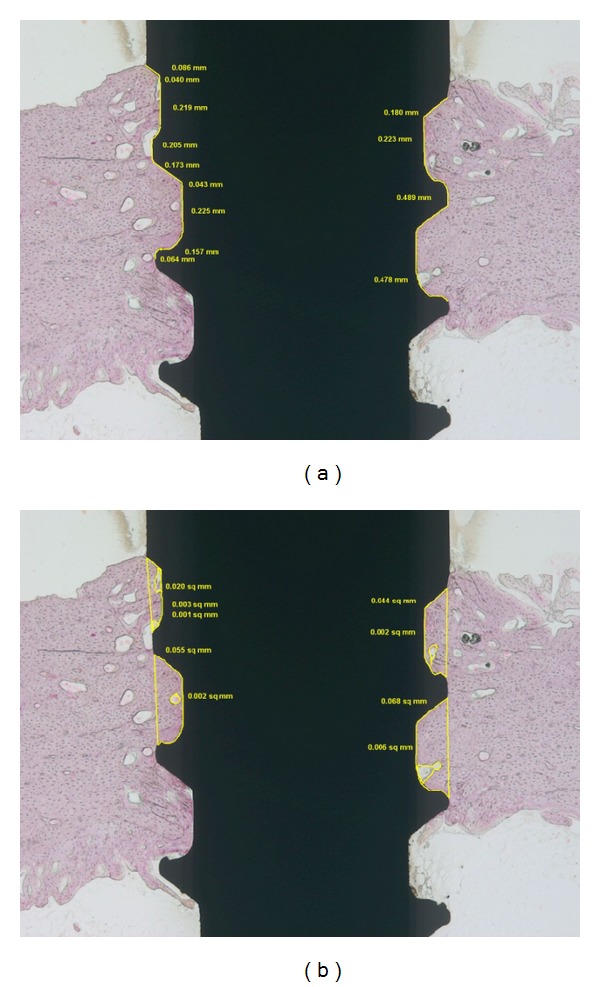
Histologic measurements. (a) Bone to implant contact, the percentage of linear surface of the implant directly contacted by mineralized bone (BIC%). (b) Bone density, the percentage of mineralized bone over the total tissue volume (BV/TV%).

Tissue specimens were prepared from 2 diabetic and 2 control rabbits. Micro-CT images (pixel size—7 *μ*m) were obtained using SkyScan-1172 high-resolution micro-CT (Skyscan, N.V., Kontich, Belgium) after fixation of specimens by 10% neutral formalin for 2 days. 3D images were reconstructed with software (CTrecon, SkyScan N.V., Kontich, Belgium). Tissue volume (TV), bone volume (BV), bone-volume fraction (BV/TV), trabecular thickness, trabecular number, and trabecular separation in 1 mm area around implants were analyzed with CTAn (SkyScan N.V., Kontich, Belgium) software. Cortical bone and bone marrow was separately analyzed (Figure 3). After micro-CT scanning, tissue specimens were fixed again in 10% neutral formalin for 2 weeks and dehydrated with ethanol series and cured specimens were cut into 220 ± 20 mu thick and were ground into 40 ± 5 mu thick using EXAKT system (KULZER EXAKT400CS, Germany) and stained with HE solution. The specimens were examined under a light microscope (BX51, OLYMPUS, Japan) with CCD camera (Diagnostic Instrument, USA). Image analysis was performed with designated software, SPOT Software V4.0 (Diagnostic Instrument, USA) and Image Pro plus (MediaCybernetics, USA) (Figure 4).

### 2.4. Statistical Analysis

Two-way ANOVA was performed to compare effects of diabetes and placement methods [17, 18]. Mann-Whitney test was used to confirm significant differences of values, bone-volume fraction, trabecular thickness, trabecular number, trabecular separation, bone to implant contact, and bone density, according to the presence of diabetes and placement methods.

## 3. Results

### 3.1. Body Weights and Blood Glucose Level

**Figure 5:**
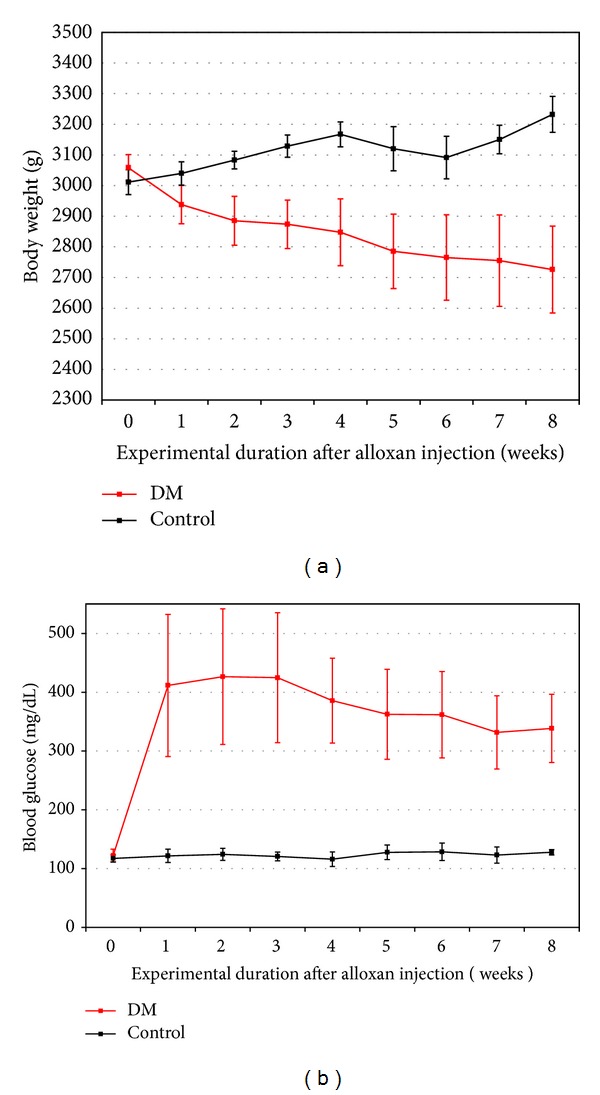
(a) Average body weights of alloxan-induced diabetic rabbit throughout the 8-week experimental period. (b) Blood glucose levels of alloxan-induced diabetic rabbit throughout the 8-week experimental period.

Diabetic rabbits showed continuous decrease in body weights. Controls showed continuous increase in weights except at four weeks when implants were placed (Figure 5(a)). High blood glucose level over 300 mg/dL was achieved after injection of alloxan and maintained throughout the experiment. Controls showed less variable blood glucose level ranging from 105 to 143 mg/dL (Figure 5(b)).

### 3.2. Comparative Analysis of Microstructure of Bone

As for bone-volume fraction and trabecular number, similar result was found in cortical bone between the controls and diabetic rabbits. In bone marrow, however, diabetic rabbits showed smaller bone-volume fraction and trabecular number than the controls. Trabecular thickness was slightly higher in both cortical bone and bone marrow between the control and diabetic groups. Diabetic rabbits had less trabecular separation in cortical bone, but more trabecular separation in bone marrow (Table 1).

### 3.3. Maximum Torque and Total Energy

When compared to the controls, maximum insertion torque was smaller in diabetic rabbits without statistical significance. In diabetic group, maximum insertion torque was higher in SD system without statistical significance. In control group, SD and ST systems showed similar results. Total insertion energy was significantly higher in SD system in both diabetic and control groups (*P* value < 0.01). Maximum removal torque and total removal energy were similar regardless of placement method and diabetics (Table 2).

### 3.4. Histomorphometric Analysis of Peri-Implant Bone

Mini-implants in this study maintained stability until the completion of the experiment and showed 100% success rate. In cortical bone, all values were similar regardless of placement method and diabetes (Table 3). Trabecular separation was significantly higher in diabetic rabbits than in controls (*P* < 0.05). Bone volume/tissue volume and trabecular thickness were smaller in a diabetic group. In both diabetic and control groups, the SD system showed higher bone volume/tissue volume and trabecular thickness. But there was no statistical significance. The number of trabecular was smaller in diabetic group than in control group. In diabetic group, trabecular was more abundant in SD system and in control group. No significant difference was found between SD and ST mini-implants (Table 4).

In the histometrical analysis, BIC was smaller in diabetic group than in control group. In controls, BIC was higher in SD system. In diabetic rabbits, BICs in SD system and ST system were similar but significantly variable and not statistically significant. Bone density (BV/TV%) was also smaller in diabetic group (Table 5).

## 4. Discussion

Diabetes is related to bone disease such as osteoporosis and bone fracture. Bone formation and osteoid volume are reduced early in the course of this disorder [19, 20]. The effect of diabetes on osseointegration, however, has not thoroughly verified, especially for orthodontic mini-implants. As use of orthodontic mini-implants is gaining popularity, studies were required to increase success rate and stability of mini-implants in increasing diabetic patients.

Stability is determined by the shape of mini-implant as well as bone quality, primary stability, and surgical protocol [19–21]. When placing mini-implants in adolescent patients, thin cortical bone, or low bone density such as maxilla, self-drilling mini-implant can enhance primary stability by condensing bone. On the contrary, when placing mini-implants in patients with thick cortical bone or high density bone such as mandible, self-drilling procedure reduced stability by inducing excessive stress to outer surface of the cortical bone [5, 22, 23]. Sowden and Schmitz reported greater bone damage when placing self-drilling mini-implants when compared with self-tapping mini-implant using scanning electron microscopy [5]. The endosteal surface of the self-drilling system showed large voids adjacent to the screw threads at the interface and microfractures of the bone at bone-implant interface [5]. Motoyoshi et al. investigated the relationship between the success rate and torque when tightening into the buccal alveolar bone of posterior regions. To improve success rate of 1.6 mm-diameter mini-implants, they recommended the placement torque to be from 5 to 10 NCm [23]. Self-drilling system and tapered mini-implants may increase primary stability, but harmful stress can be generated if the torque is excessive [24, 25].

In micro-CT image analysis, cortical bone did not show significant difference between SD and ST groups and diabetic and control groups. In bone marrow, however, amount and thickness of trabecular bone was decreased significantly in this study. This coincides with the previous study that reported advanced trabecular bone resorption in a diabetic state [26]. Similar result was found in surrounding bone as well. In cortical bone, no significant difference was observed between the controls and the diabetic group as well as self-drilling and self-tapping systems. More significant difference, however, was found in bone marrow. The controls showed higher bone-volume fraction, trabecular thickness, and trabecular number than the diabetic group. In both groups, the self-drilling system was higher in bone-volume fraction, trabecular thickness, and trabecular number than the self-tapping system but there were no statistical differences. The diabetic group showed higher trabecular separation than the controls and the difference was significant. From these findings, it can be assumed that placing mini-implants in area where there is more cortical bone will be more advantageous in diabetic patients.

Bone to implant contact (BIC%) and bone density (BV/TV%) were smaller in diabetic state than the controls. This resulted from reduced direct bone contact due to decreased total amount of bone in diabetic rabbits. When using the self-drilling system, however, it showed more bone contact between mini-implant and surrounding bone. This concurs with the previous studies by Heidemann et al. [4, 7].

Maximum insertion torque was less in diabetic group. It can be assumed that the mechanical property of cortical bone was weakened due to diabetes, however, no statistical difference was found. In this study, the results came from four weeks after placement. If this period increases, the effect of diabetes on bone will increase as well. The self-drilling and self-tapping systems showed a similar result in maximum insertion torque in both controls and diabetic group. This result did not match with the expectation that the self-drilling system, which has no predrilling procedure, will have higher maximum insertion torque than the self-tapping system. Total insertion energy is the energy absorbed by bone from the beginning to the maximum torque value of mini-implant insertion. It can be calculated by measuring torque value continuously during insertion [27]. In both controls and diabetic group, total insertion energy was higher in SD system than STsystem (*P* < 0.001). This can be considered that more energy was absorbed into surrounding bone when using SD system. There was no significant difference between diabetic and control groups in maximum removal torque and total removal energy, regardless of placement system. Small sample size due to difficulties of maintaining diabetic state and variability of measurements make it difficult to confirm the statistical significance. Therefore, further studies with more animals and longer duration of diabetic condition are required. Moreover, further study needs to be conducted in regard to potential effect of loading on these mini-implants, since they will be loaded ultimately.

## 5. Conclusions

In this animal study, higher bone to implant contact ratio and bone density were observed through the self-drilling method than self-tapping method in controls and diabetic rabbits. This result had no statistical significance, but it can be assumed that bone was damaged or deformed due to absorbed energy. In this study, diabetes was induced through medication with animals; thus it might differ from the actual responses in human body. However, it can be concluded that the presence of diabetes and placement system do not affect the initial stability of orthodontic mini-implants.

## Figures and Tables

**Table 1:** Three-dimensional bone microstructure analysis in cortical and bone marrow area.

	Group	Area	
		Cortical area	Marrow area
		(Mean ± SD)	(Mean ± SD)
Bone volume/tissue volume (%)	DM	96.13 ± 3.79	0.11 ± 0.16
	Control	93.88 ± 0.59	1.42 ± 1.39

Trabecular thickness (mm)	DM	0.13 ± 0.04	0.04 ± 0.01
	Control	0.17 ± 0.07	0.06 ± 0.02

Trabecular number (1/mm)	DM	7.68 ± 2.15	0.02 ± 0.03
	Control	6016 ± 2087	0.18 ± 0.14

Trabecular separation (mm)	DM	0.03 ± 0.00	0.97 ± 0.01
	Control	0.05 ± 0.01	0.80 ± 0.21

**Table 2:** Maximum torque (Ncm) and total energy (J) during insertion and removal.

	Group	Type of pilot drilling		Significance
		Self-drilling	Self-tapping	
		(Mean ± SD)	(Mean ± SD)	
Maximum insertion torque (Ncm)	DM	9.88 ± 1.30	9.38 ± 2.76	NS
	Control	10.75 ± 1.20	10.75 ± 1.83	

Total insertion energy (J)	DM	3.56 ± 1.01	2.27 ± 0.63	Self-drilling > self-tapping
	Control	3.42 ± 0.54	2.55 ± 0.47	

Maximum removal torque (Ncm)	DM	4.44 ± 1.21	4.28 ± 1.68	NS
	Control	4.13 ± 1.38	4.13 ± 2.03	

Total removal energy (J)	DM	0.52 ± 0.15	0.50 ± 0.08	NS
	Control	0.45 ± 0.10	0.44 ± 0.13	

Two-way ANOVA was calculated.

**Table 3:** Three-dimensional bone microstructure analysis in cortical bone area.

	Group	Type of pilot drilling		Significance
		Self-drilling	Self-tapping	
		(Mean ± SD)	(Mean ± SD)	
Bone volume/tissue volume (%)	DM	95.54 ± 5.18	93.96 ± 5.93	NS
	Control	95.87 ± 4.24	98.00 ± 2.17	

Trabecular thickness (mm)	DM	0.16 ± 0.07	0.19 ± 0.10	NS
	Control	0.24 ± 0.10	0.28 ± 0.14	

Trabecular number (1/mm)	DM	6.91 ± 2.78	5.95 ± 2.81	NS
	Control	4.46 ± 1.39	4.32 ± 2.17	

Trabecular separation (mm)	DM	0.04 ± 0.01	0.04 ± 0.01	NS
	Control	0.04 ± 0.01	0.03 ± 0.01	

Mann-Whitney test was used.

**Table 4:** Three-dimensional bone microstructure analysis in bone marrow area.

	Group	Type of pilot-drilling		Significance
		Self-drilling	Self-tapping	
		(Mean ± SD)	(Mean ± SD)	
Bone volume/tissue volume (%)	DM	6.05 ± 2.81	4.91 ± 0.96	NS
	Control	8.24 ± 2.23	7.90 ± 3.04	

Trabecular thickness (mm)	DM	0.09 ± 0.01	0.08 ± 0.00	NS
	Control	0.10 ± 0.00	0.09 ± 0.02	

Trabecular number (1/mm)	DM	0.66 ± 0.22	0.61 ± 0.10	NS
	Control	0.83 ± 0.22	0.84 ± 0.30	

Trabecular separation (mm)	DM	0.91 ± 0.08	0.86 ± 0.04	NS
	Control	0.80 ± 0.05	0.81 ± 0.05	

Mann-Whitney test was used.

**Table 5:** Comparison of BIC% and BV/TV% values between groups.

	Group	Type of pilot drilling		Significance
		Self-drilling	Self-tapping	
		(Mean ± SD)	(Mean ± SD)	
BIC%	Control	86.21 ± 8.18	82.72 ± 9.84	NS
	DM	73.03 ± 13.55	73.16 ± 28.89	

BV/TV%	Control	92.69 ± 2.86	90.05 ± 5.55	NS
	DM	79.34 ± 12.07	70.02 ± 31.21	
